# The saga of dyssynchrony imaging: Are we getting to the point

**DOI:** 10.3389/fcvm.2023.1111538

**Published:** 2023-03-31

**Authors:** Elena Galli, Vincent Galand, Virginie Le Rolle, Marion Taconne, Adrien Al Wazzan, Alfredo Hernandez, Christophe Leclercq, Erwan Donal

**Affiliations:** Department of Cardiology and Vascular Disease, Univ Rennes, CHU Rennes, Inserm, LTSI – UMR 1099, Rennes, France

**Keywords:** cardiac imaging, cardiac resynchronization therapy, left ventricular dyssynchrony, heart failure, left ventricular remodelling

## Abstract

Cardiac resynchronisation therapy (CRT) has an established role in the management of patients with heart failure, reduced left ventricular ejection fraction (LVEF < 35%) and widened QRS (>130 msec). Despite the complex pathophysiology of left ventricular (LV) dyssynchrony and the increasing evidence supporting the identification of specific electromechanical substrates that are associated with a higher probability of CRT response, the assessment of LVEF is the only imaging-derived parameter used for the selection of CRT candidates.

This review aims to (1) provide an overview of the evolution of cardiac imaging for the assessment of LV dyssynchrony and its role in the selection of patients undergoing CRT; (2) highlight the main pitfalls and advantages of the application of cardiac imaging for the assessment of LV dyssynchrony; (3) provide some perspectives for clinical application and future research in this field.

**Conclusion:**

the road for a more individualized approach to resynchronization therapy delivery is open and imaging might provide important input beyond the assessment of LVEF.

## 1. Introduction

Cardiac resynchronisation therapy (CRT) has an established role in the management of patients with heart failure (HF), a severely reduced left ventricular ejection fraction (LVEF <35%) and a QRS > 130 msec, who remain symptomatic despite an optimized medical therapy (1).

However, 30%–40% of patients receiving CRT according to recommendations (1) do not experience significant LV reverse remodelling and improvement in LVEF (2).

In the last 20 years, significant efforts have been made for identifying imaging-derived parameters able to disclose the electromechanical substrates associated with CRT response, substantially increasing the knowledge of the pathophysiological mechanisms of LV dyssynchrony and potentially contributing to the improvement in the selection of CRT candidates.

This paper aims to 1) provide an overview of the evolution of cardiac imaging for the assessment of LV dyssynchrony and its role in the selection of patients undergoing CRT; 2) highlight the main pitfalls and advantages of the application of cardiac imaging for the assessment of LV dyssynchrony; 3) provide some perspectives for clinical application and future research in this field.

## 2. Pathophysiology of left ventricular dyssynchrony

The loss of a nearly simultaneous LV contraction in patients with typical left bundle branch block and left ventricular dyssynchrony is associated with the presence of early septal activation, starting at low LV pressure, which does not contribute to LV ejection and stretches the lateral wall. The stretch of the lateral wall further delays shortening and causes a vigorous activation against a locally increased preload (3). This alternation of activation and stretch of opposite LV walls seen in the dyssynchronous heart promotes local modifications of the LV function at the molecular and cellular level (4), with redistribution of myocardial blood flow and oxygen uptake (5), and the development of differences in septal-to-lateral wall thickness (6, 7). This deleterious pathophysiological process can be reversed in the case of successful CRT and is associated with positive LV reverse remodelling. However, in some patients, LV dyssynchrony is not attributable to specific electromechanical substrates responsive to CRT, but to other causes such as ischemia or left ventricular hypocontractility, which are associated with poor or absent CRT response (8–10). In the following paragraphs we will try to underscore the progress of myocardial imaging in disclosing the specific mechanisms associated with LV dyssynchrony in order to improve patients' selection and CRT success.

## 3. Imaging for the selection of CRT candidates

The mechanism of action of CRT in patients with HF and widened QRS are far to be fully elucidated. This incomplete understanding of the pathophysiology of the disease can explain the variety of potential responses to CRT going from a spectacular LV reverse remodelling, often referred to as “CRT super-response” (11) to milder effects, and even worsening of LV function (12–14). This is probably because CRT is primarily designed to correct the conduction disorders corresponding to a widened QRS, but with the main aim to improve LV mechanical efficiency. In this context, the assessment of LV mechanical dyssynchrony through cardiac imaging has been proposed as an additive criterion to select CRT recipients.

### 3.1. Assessment of left ventricular opposite wall delay

The initial imaging studies on LV mechanical dyssynchrony were focused on the measure of opposite wall delay by different echocardiographic modalities.

**Figure 1:**
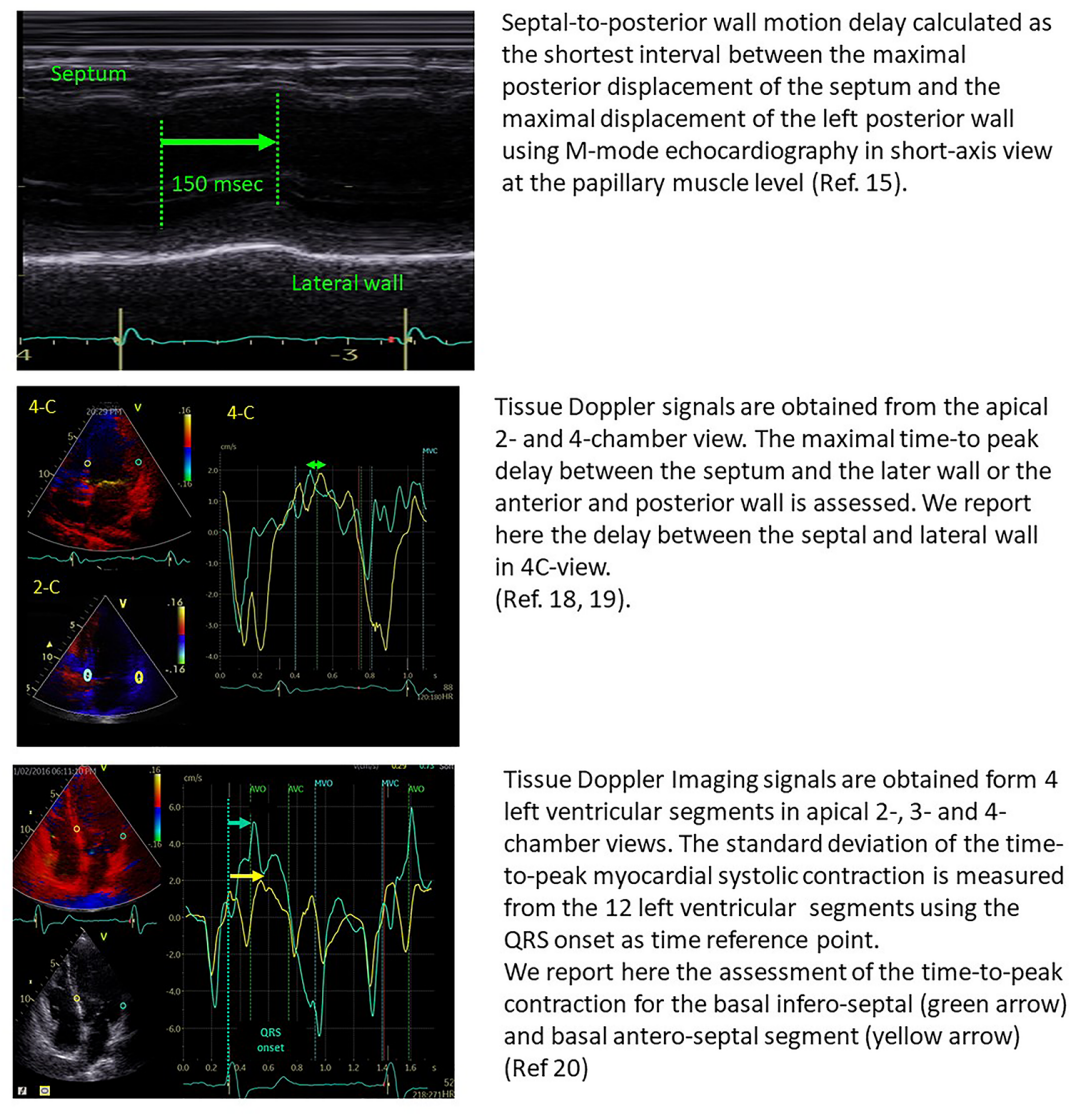
Examples of the estimation of opposite wall delay in CRT candidates according to the main methods described in the literature. Upper panel: Pitzalis’ method; Middle panel: Bax's method; Lower panel: Yu's method.

In 24 patients with heart failure, Pitzalis et al. showed that a septal-to-posterior motion delay ≥130 msec was able to predict LV reverse remodelling, with a positive predictive value of 80% and an accuracy of 85% (15). However, this approach was not suitable for patients with previous anterior or septal infarction and was plagued by poor temporal resolution (16). Successive studies, therefore, focused on tissue Doppler imaging and speckle tracking echocardiography for the assessment of LV dyssynchrony, by the estimation of opposite wall delay (17), or by focusing on the difference in peak systolic velocities of different myocardial regions (18–20) (Figure 1). Despite all these studies being able to show the good performance of echo-derived parameters for the prediction of CRT response in small, retrospective cohorts, the multicentric PROSPECT trial did not confirm the applicability of echocardiographic measures of dyssynchrony for the selection of CRT candidates (2).

These disappointing results are attributable to the poor reproducibility of the echo-derived analysis of dyssynchrony parameters (21), but also to the fact that the assessment of opposite wall delay is not able to disclose the complex pathophysiology related to CRT response. Computer simulation studies and clinical data have shown that there are several potential mechanisms associated with LV dyssynchrony, including electrical activation delay, regional differences in contractility and myocardial scar (9), so that only specific electromechanical substrates associated with a widened QRS are amenable to be corrected by CRT (Table 1).

**Table 1:** Summary of the main cited studies providing an overview of the role of imaging-derived parameters modalities in the field of CRT.

Assessment of LV opposite wall delay				
Authors/*Journal – year/*Ref	Population	Endpoints	Imaging parameters	Results
Pitzalis MV et al. *J Am Coll Cardiol 2002.* (15)	24 pts LVEF 24 ± 5%, QRS width 169 ± 16 msec, 20% ischemic	LVESV reduction > 15% at 6 months FU	Septal-to-lateral wall delay > 130 msec	AUC 0.95, PPV 80%, Sp 63%, Accuracy 85% for the prediction of LV reverse remodelling
Marcus GM et al. *J Am Coll Cardiol 2005.* (16)	79 pts LVEF 22 ± 7%, QRS width 159 ± 27 msec, 72% ischemic	LVESV reduction > 15% at 6 months FU	Septal-to-lateral wall delay > 130 msec	Se 24%, Sp 66%, PPV 29%, NPV 61% for the prediction of LV reverse remodelling
Søgaard P et al. *J Am Coll Cardiol 2002.* (17)	25 pts LVEF 29 ± 7%, QRS width 189 ± 23 msec, 44% ischemic	LVEF and GSCA improvement	Basal segment delayed contraction at TDI	Prediction of LVEF improvement: *r* = 3.1, *p* < 0.01; Prediction of GSCA improvement: *r* = 3.3, *p* < 0.01
Gorcsan J et al. *Am J Cardiol 2004.* (18)	29 patients LVEF 26 ± 6% %, QRS width 175 ± 34 msec, 44% ischemic	Acute LV stroke volume increase >15%	Antero-septum to posterior wall delay > 65 msec at TDI	Se 87%, Sp 100% for the prediction of acute of LV reverse remodelling
Bax JJ et al. *J Am Coll Cardiol 2004.* (19)	86 pts LVEF <40% QRS > 140 msec, 55% ischemic	1) LVESV reduction > 15% at 6 months FU 2) Clinical improvement	LV opposite wall delay > 65 msec at TDI	Se 92% and Sp 92% for the prediction of LV reverse remodelling; Se 80%, Sp 80% for the prediction of clinical improvement
Yu CM *Am J Cardiol 2003*. (20)	30 pts LVEF 23 ± 7%, QRS width 150 ± 18 msec, 40% ischemic	LVESV reduction > 15% at 6 months FU	Standard deviation of the time-to-peak systolic strain of 12 LV segments	Predictor of LV reverse remodelling (*β* = −1.54, *p* 0.007). Se and Sp 100% for the prediction of CRT response.
**Assessment of LV strain dynamics and mechanical dyssynchrony**				
Lumens J et al. *Circ Cardiovasc Imaging 2015.* (9)	191 pts LVEF 24 ± 6%, QRS width 159 ± 27 msec, 60% ischemic	Primary: HF hospitalization or overall death Secondary: overall deart, heart transplant or LV-assisted device	Systolic stretch index (SSI)	SSI ≥ 9.7% is an independent predictor of the primary endpoint (HR = 0.32; 95% CI: 0.19–0.53; *p* < 0.001) and secondary endpoint (HR = 0.28; 95% CI: 0.15–0.55, *p* < 0.001)
Lim P et al. *Eur J Heart Fail 2011.* (22)	189 pts LVEF 26 ± 8%, QRS width 151 ± 34 msec, 33% ischemic	LVESV reduction > 15% at 6 months FU	Strain delay index	AUC 0.80, Se 92%, Sp 65%, PPV 80%, NPV 84% for the prediction of LV reverse remodelling
Bernard A et al. *J Am Soc Echocardiogr 2015*. (23)	130 patients LVEF 27 ± 6%, QRS width 162 ± 23 msec, 38% ischemic	LVESV reduction > 15% at 6 months FU	Difference of strain integrals between AVC and strain peak (Diff_Int_)	Higher Diff_int_ in the lateral wall in responders
Risum N et al. *J Am Coll Cardiol 2015.* (24)	208 pts LVEF <35%, QRS width > 120 msec, 58% Ischemic	Cumulative death, left ventricular assist device, or heart transplantation	Typical vs. atypical LBBB strain patters	Absence of “Typical” LBBB strain pattern is an independent prognostic predictor: HR = 3.1; 95% CI: 1.64 to 5.88, *p* < 0.005
Gorcsan J et al. *JACC Cardiovasc Imaging 2018.* (25)	442 pts LVEF 25 ± 7%, QRS width 155 ± 21 msec, 47% Ischemic	Primary: HF hospitalization or death; Secondary: death	Systolic stretch index	SSI < 3.1% is an independent predictor of the primary endpoint (HR = 2.17; 95% CI: 1.45 to 3.24, *p* < 0.001) and secondary endpoint (HR = 4.06; 95% CI: 1.95 to 8.45, *p* < 0.001)
Parsai C et al. *Eur Heart J 2009*. (26)	161 pts LVEF 24 ± 7%, QRS width 156 ± 21 msec, 51% Ischemic	LVESV reduction > 10% at 6 months FU	Septal flash (SF)	Reversal of septal flash after CRT is associated with 100% reverse remodelling
Stankovic I et al. *Eur Heart J Cardiovasc Imaging.* 2017 (27)	1,060 patients LVEF 27 ± 7%, QRS width 170 ± 29 msec, 43% Ischemic	1) LVESV reduction > 15% at 6 months FU 2) Overall death	Septal flash/Apical rocking (ApR)	Reverse remodelling prediction: SF: Se = 70%, Sp = 74%, Accuracy = 77% ApR: Se = 84%, Sp = 79%, Accuracy = 82% Mortality prediction: SF: HR = 0.45, 95% CI: 0.34– 0.61, *p* < 0.0001 ApRock: HR = 0.40, 95% CI: 0.30–0.53, *p* < 0.0001)
Marsan NA et al. *European Heart Journal 2009.* (28)	35 patients LVEF 24 ± 8%, QRS width 145 ± 33 msec, 57% Ischemic	LVESV reduction > 15% at 6 months FU	16-segment time-to-maximum radial wall thickness assessed at CMR (16-SD); scar extent	Independent predictors of LV reverse remodelling 16-SD: OR = 6.3, 95% CI 3.1–9.9, *p* < 0.001 Scar: OR = 0.52, 95% CI 0.43–0.87, *p* < 0.001
**Assessment of myocardial contractility and global left ventricular function**				
Parsai C et al. *Eur Heart J 2009*. (29)	52 pts LVEF 24 ± 7%, QRS width 145 ± 24 msec, 44% Ischemic	LVESV reduction > 10% at 7 ± 1 months FU	DSE-induced SF	Prediction of LV reverse remodelling (R = 0.6, *p* < 0.0001)
Ciampi Q et al. *European Journal of Heart Failure 2009.* (30)	69 pts LVEF 27 ± 6%, QRS width 150 ± 27 msec, 55% Ischemic	LVESV reduction > 15% at 6 months FU	Contractile reserve at DSE	Best predictor of LV reverse remodelling: OR = 6.2, 95% CI: 1.4–27.6, *p* < 0.015
Delgado-Montero A et al. *Circ Cardiovasc Imaging 2016.* (31)	205 pts LVEF 24 ± 6%, QRS width 157 ± 26 msec, 63% Ischemic	Primary: death, circulatory support, or transplant Secondary: HF hospitalization or death	GLS, GCS	Independent predictors of the primary endpoint: GLS > −9%: HR = 2.91; 95% CI: 1.88–4.49, *p* < 0.001 GCS > −9%: HR = 3.73; 95% CI: 2.39–5.82, *p* < 0.001) Independent predictors of the secondary endpoint: GLS > −9%: HR = 2.10; 95% CI: 1.45–3.05, *p* < 0.001 GCS > −9%: HR = 3.25; 95% CI: 2.23–4.75, *p* < 0.001.
Khidir MJH et al. *Heart Rhythm 2018.* (32)	829 pts LVEF 27 ± 8%, QRS width 149 ± 30 msec, 60% Ischemic	Primary: overall death, heart transplantation, and LV assist device implantation Secondary: ventricular arrhythmias or appropriate ICD therapy	GLS	Independent predictor of the primary endpoint (HR = 1.075, 95% CI: 1.020–1.133, *p* = 0.007) but not of the secondary endpoint
van der Bijl P et al. *Eur Heart J Cardiovasc Imaging 2019.* (33)	1,185 pts LVEF 27 ± 8%, QRS width 155 ± 35 msec, 56% Ischemic	Overall death	≥15%↓LVESV and/or ≥5%↑|GLS| at 6-month FU	Independent predictors of overall death: ≥15%↓LVESV and ≥5% ↑|GLS|: HR = 0.47; 95% CI: 0.31–0.71, *p* < 0.001; ≥15%↓LVESV and <5%↑|GLS| or <15%↓LVESV and ≥5%↑|GLS: HR = 0.57, 95% CI: 0.47–0.71, *p* < 0.001
**Assessment of scar extension and localisation**				
Delgado V et al. *Circulation 2011.* (34)	397 pts LVEF 25 ± 7%, QRS width 155 ± 33 msec, 100% Ischemic	HF hospitalization and overall death	LV radial dyssynchrony, discordant lead position, scar in the targeted segment	LV dyssynchrony: HR = 0.994, 95% CI: 0.992–0.998, *p* = 0.001 Discordant lead: HR = 2.086, 95%: 1.336–3.258, *p* = 0.001 Scar in the targeted segment: HR = 2.913, 95% CI: 1.740–4.877, *p* < 0.001
Khan FZ et al. *J Am Coll Cardiol 2012.* (35)	220 pts LVEF < 30%, QRS width > 130 msec, 56% ischemic	Primary: LVESV reduction >15% at 6 months FU. Secondary: overall death and HF hospitalization	LV lead position at the latest activated viable segment (Target group vs. non-target group)	Significant *Δ*LVESV in the target group vs. non-target group: −46 ± 33 vs. −26 ± 23 ml and better prognosis (log-rank *p* = 0.031)
Bose A et al. *J Cardiovasc Electrophysiol 2014.* (36)	160 patients LVEF 25 ± 7%, QRS width 159 ± 28 msec, 100% ischemic	Primary: HF hospitalization and overall death	Myocardial substrate at the site of LV lead by SPECT-MPI	Independent predictors of prognosis: scar at the LV lead: HR = 2.07, 95%CI: 1.14–3.74, *p* = 0.015 ischemia and scar at the LV lead: HR = 2.03 95% CI: 1.03–4.0, *p* = 0.040
Adelstein EC et al. *European Heart Journal 2011.* (37)	624 patients LVEF 24 ± 6%, QRS width 169 ± 33 msec	Primary: overall death; cardiac transplant, or mechanical circulatory support	Scar assessed at SPECT	Independent predictor of prognosis: Scar: HR = 1.8, 95% CI: 1.3–2.5, *p* < 0.001, unsuccessful LV lead implant: HR = 2.4, 95% CI: 1.4–4.2, *p* < 0.001)
Aalen JM et al. Eur Heart J 2020. (8)	220 pts LVEF <35%, QRS width 167 ± 21 msec, 35% ischemic	Primary: LVESV reduction >15% at 6 months FU. Secondary: overall death and heart transplantation	Septal-to-lateral wall work difference; scar localisation at MRI	Septal-to-lateral wall work difference (B = −0.011, *p* < 0.0001) and septal scar (*β* = 0.42, *p* = 0.029) are the main determinants of LV reverse remodelling and prognosis (AUC 0.88, Se 86%, Sp 84%, Accuracy 85%)
**Importance of a comprehensive evaluation of cardiac function**				
Moss AJ et al. *N Engl J Med* 2009 (38)	1,820 patients LVEF < 30%, QRS width > 130 msec, 55% ischemic	Overall death, HF hospitalisation	LV size and function	
Carluccio E et al. *JACC Cardiovasc Imag* 2011. (39)	78 pts LVEF 26 ± 6%, QRS width 165 ± 30 msec, 33%ischemic	1) Improvement in LVEF 2) Cardiac events	LV size	Indexed LVESVI > 103 ml/m^2^ is an independent predictor of cardiac events (HR = 2.53, 95% CI: 1.17–5.44, *p* = 0.017)
Galli E et al. *Int J Cardiol 2021.* (40)	193 LVEF 28 ± 8%, QRS width 167 ± 21 msec, 33% ischemic	Overall death, heart transplantation, LV-assisted device implantation	Diastolic dysfunction degree	Grade I diastolic dysfunction portends a better prognosis (HR = 0.37, 95%CI: 0.14–0.96; log-rank *p* vs. grade II-III diastolic dysfunction <0.0001)
Kuperstein R et al. *Circ Heart Fail 2014.* (41)	1,785 pts LVEF < 35%, QRS width > 130 msec	HF hospitalisation or overall death	LA volume	LA volume > 52 ml/m^2^ is an independent prognostic predictor: HR = 1.69, 95% CI: 1.35–2.11, *p* < 0.001, log-rank *p* < 0.001
Galli E et al. *Eur Heart J Cardiovasc Imaging 2022.* (42)	221 pts LVEF < 35%, QRS width > 165 ± 26 msec, ischemic 33%	LVESV reduction >15% at 6 months FU LVEDV reduction > 10% at 6 months FU	LA reservoir strain	Independent predictor of systolic (*β*-0.14, *p* = 0.049) and LV diastolic remodelling (*β* = −0.17, *p* = 0.001)
Damy T et al. *J Am Coll Cardiol 2013.* (43)	688 pts from CARE-HF, 345 receiving CRT Median LVEF 24 (21–28) %, QRS > 130 msec, 33% ischemic	Overall mortality	TAPSE	TAPSE <17 mm is an independent prognostic predictor (log-rank *p* < 0.0001) in both CRT and medical treated patients
Rapacciuolo A et al. *Clin Cardiol 2016.* (44)	227 pts LVEF28 ± 6%, QRS width > 162 ± 26 msec, ischemic 41%	LVESV reduction >15% at 6 months FU	TAPSE	TAPSE > 17 mm is an independent predictor of LV remodelling; Se 68%, Sp 54%; OR = 1.97, 95% CI: 1.03–3.80, *p* < 0.05)

ApR, apical rocking; AUC, area under the curve; AVC, aortic valve closure; CI, confidence interval; CMR, cardiac magnetic resonance; CRT, cardiac resynchronization therapy; DSE, dobutamine stress echocardiography; Diff_int_, difference of strain integrals between AVC and strain peak; FU, follow-up; GCS, global circonferential strain; GLS, global longitudinal strain; GSCA, global systolc contraction amplitude; HF, heart failure; HR, hazard ratio; ICD, intracardiac defibrillator; LA, left atrium; LBBB, left bundle branch block; LV, left ventricle; LVEF, left ventricular ejection fraction; LVEDV, left ventricular end-diastolic volume; LVESV, left ventricular end-systolic volume; MPI, myocardial perfusion imaging; 6 MWT, six-minute = walking test; NYHA, New York Heart Association functional class; NPV, negative predictive value; OR, odds ratio; PPV, positive predictive value; Se, sensitivity; SF, septal flash; SPECT, single-photon emission computed tomography; Sp, specificity; SSI, systolic stretch index; SV stroke volume; TAPSE, tricuspidannular plane systolic excursion; TDI, tissue Doppler imaging.

### 3.2. Assessment of strain curves dynamics

**Figure 2:**
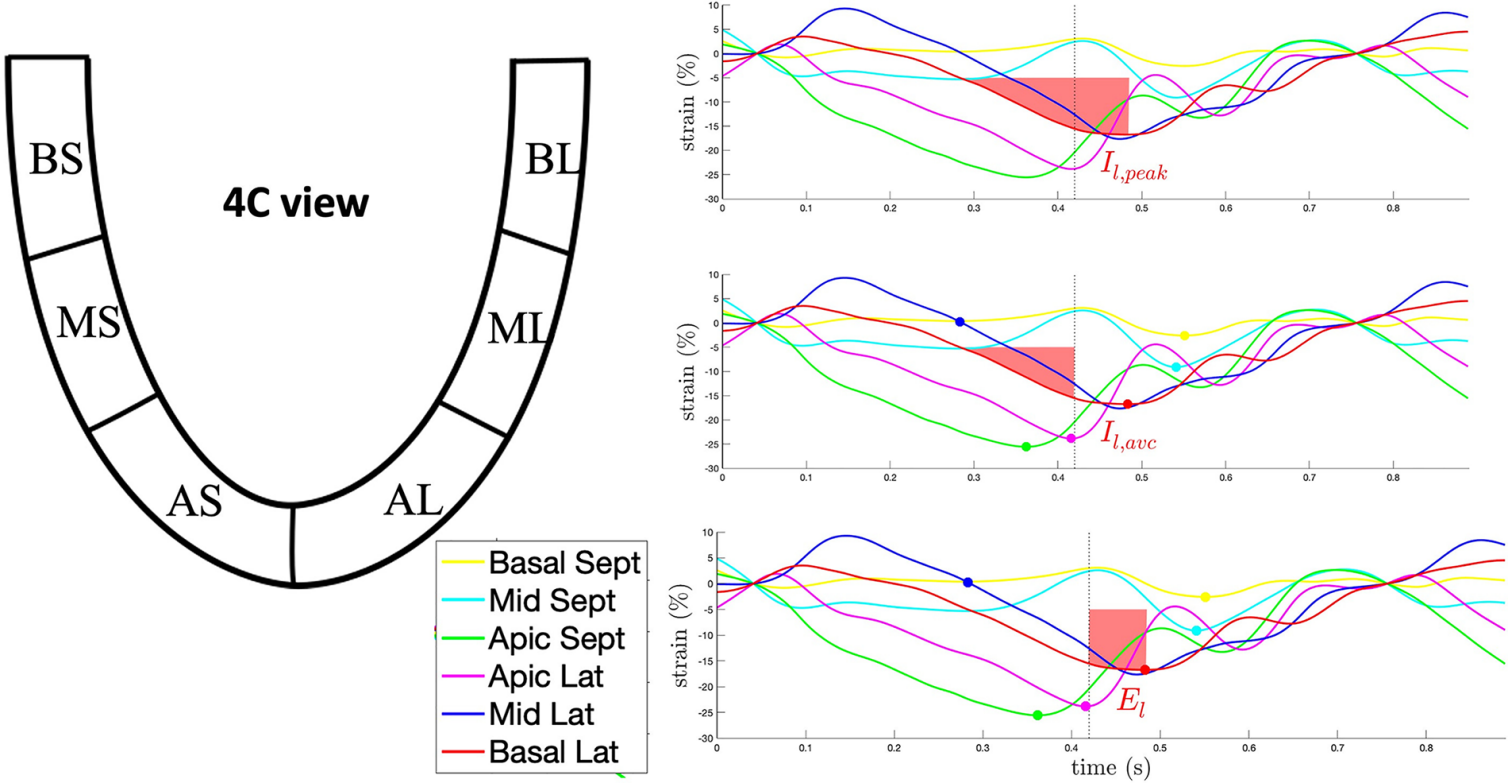
Example of the semi-quantitative analysis of the strain curve dynamics through the calculation of strain integrals for the basolateral segment in 4-chamber view. Upper panel: the red-shaded area represents the integral to strain peak (I*_l,peak_*). Middle panel: the red-shaded area represents the integral to aortic valve closure (I*_l,AVC_*). Lower panel: the red-shaded area represents E*_l_*, the difference between I*_l,peak_* and I*_l,AVC_*, corresponding to the waste energy due to the delayed lateral wall contraction. AL, apico-lateral segment; AS, apico-septal segment; BL, basal-lateral segment; BS, basal-septal segment; ML, mid-lateral segment; MS, mid-septal segment.

The careful analysis of the dynamics and morphology of strain curves more than the evaluation of timings can provide further insight into the mechanisms of LV dyssynchrony and ease the identification of specific deformation patterns associated with CRT response. In 189 CRT candidates, the Multicentre study using strain delay index for predicting response to cardiac resynchronization therapy (MUSIC) study showed that in the case of preserved contractility and significant dyssynchrony, LV segments present a higher difference between the peak and end-systolic strain, which is a measure of segmental wasted energy. The global LV wasted energy is referred to as strain delay index (SDI) and has been shown to predict CRT response with an AUC of 0.88, a sensitivity of 92% and a specificity of 65% (22). A similar approach for the assessment of strain dynamics relies upon the semi-automatic analysis of strain integrals. Bernard et al. reported a significant heterogeneity of strain integrals in CRT-responders compared to non-responders. Moreover, these authors showed that the difference of strain integrals measured at strain peak and aortic valve closure, which represent the wasted energy of the LV myocardium, is higher in CRT-responders and is corrected by successful CRT (23) (Figure 2).

**Figure 3:**
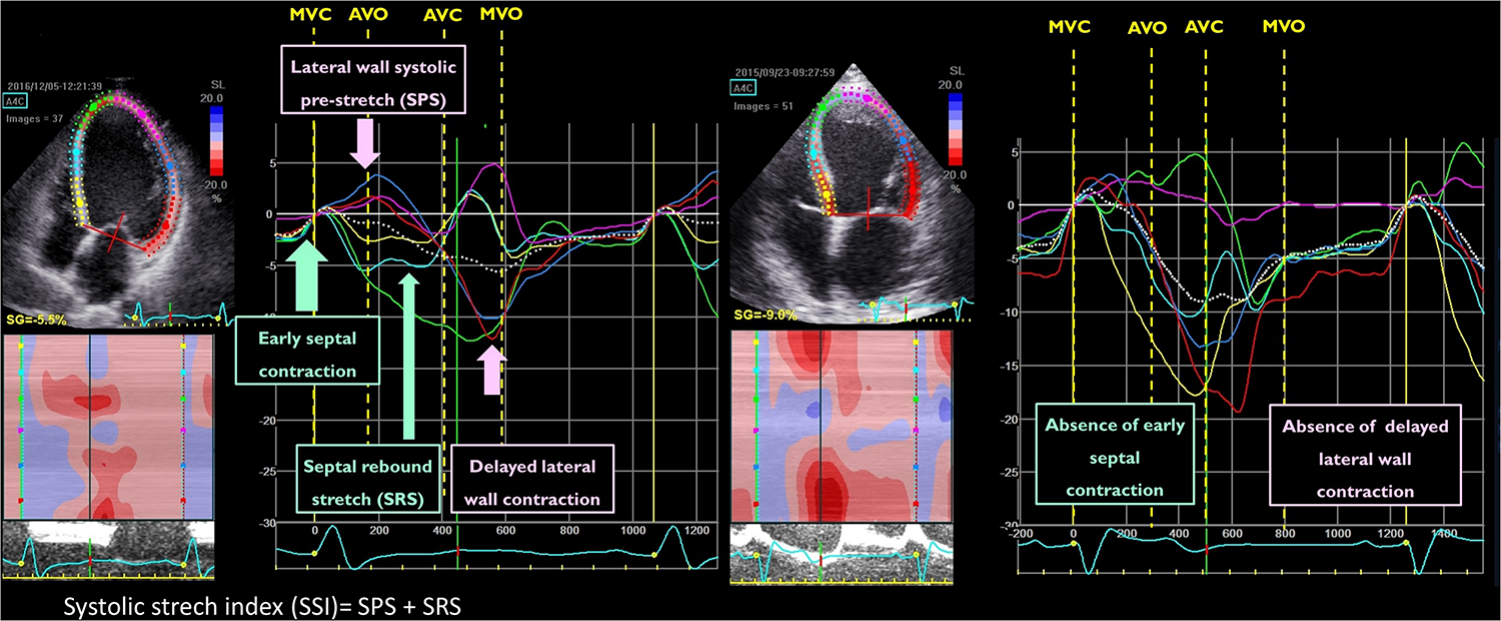
Example of the qualitative analysis of strain curves in a patient with a “typical” left bundle branch block (left panel) and “atypical” left bundle branch block (right panel). The systolic stretch index (SSI) quantification is exemplified in the same picture (left panel).

Starting from the visual analysis of strain curves obtained in a 4-chamber view in the septal and lateral wall, Risum et al. have shown that the “typical left bundle branch block” (LBBB) strain pattern is characterized by an early shortening of the septal wall, before the opening of the aortic valve, with concomitant stretch in the lateral wall. This early septal activation is followed by immediate lengthening (rebound stretch) and causes a delayed lateral wall peak contraction (Figure 3). This specific activation pattern has been shown to improve the prediction of LV reverse remodelling and prognosis after CRT (24). Computer simulation studies have demonstrated that the progressive decline in LV contractility is associated with a loss of the septal-to-lateral wall interplay typical of LBBB, such explaining the poor CRT-response in these patients (45). The sum of the posterolateral systolic prestretch and septal systolic rebound stretch, referred to as systolic stretch index (SSI) can be used to quantify the electromechanical substrate of dyssynchronous heart and has been shown to be associated with both CRT-response and prognosis (9, 25). Interestingly, an SSI > 2.6% was able to predict death or HF hospitalisation (HR: 2.08; 95% CI: 1.27 to 3.41, *p* = 0.004) and overall survival (HR 5.08; 95% CI: 1.94 to 13.31, *p* < 0.001) also in patients with a QRS width 120 to 149 ms or non-LBBB morphology (Figure 3).

The localisation of myocardial scar can also impact septal motion patterns in LBBB. In an experimental study, Aalen et al. have shown that the presence of lateral wall scar leads to the loss of septal rebound stretch, whereas extensive anterior ischemia increases rebound stretch (10), suggesting that scar localisation together with septal motion should be taken into account in the evaluation of LV dyssynchrony (Table 1).

### 3.3. The visual assessment of dyssynchrony: septal flash and apical rocking

Another approach to assess the unique contraction pattern typical of LBBB relies on the analysis of the contraction of opposite wall looking for the presence of septal flash (SF) (26) and/or apical rocking (ApR) (46).

The SF corresponds to the early septal thickening/thinning during the isovolumic systole and can be easily detected by M-mode parasternal long-axis view of by tissue Doppler imaging in short or long parasternal long axis view (26, 47). The ApR corresponds to apical transverse motion due to the early septal contraction followed by the delayed activation of the lateral wall (46). Both these parameters have shown to portend LV residual contractility in CRT-candidates (48), to predict CRT-response and long-term survival after CRT implantation, and to improve the prognostic stratification of guideline-based patients' selection (27).

Moreover, these motion patterns have proven to predict CRT-response in patients with chronic right ventricular pacing needing a pacemaker upgrade to CRT (49).

However, the correct identification of septal flash and apical rocking in CRT candidates is strictly limited by the experience of the echo-reader. In a small monocentric study, Mada et al. have shown that the semi-automatic detection of these specific contraction patterns by speckle tracking echocardiography performed better than novice echo-readers for the identification of SF/ApR (50) (Table 1).

Despite the visual assessment of LV dyssynchrony is often performed by echocardiography, other imaging modalities such as cardiac magnetic resonance (CMR) and single photon emission computed tomography (SPECT) might be used to detect LV mechanical discoordination. Both these imaging modalities allow the contemporary assessment of scar localization and viability, and they might provide an interesting approach for the evaluation of CRT candidates before device implantation (28, 51, 52). However, these imaging modalities are plagued by a poor temporal resolution which can impact the refined assessment of LV dyssynchrony, thus favoring echocardiography for this specific purpose (53).

### 3.4. Assessment of myocardial contractility and global left ventricular function

Myocardial contractility and scar localisation are two important parameters associated with CRT-response.

Dobutamine stress echocardiography (DSE) can be used to identify the presence of contractile reserve and LV dyssynchrony in CRT-candidates (29, 30). In 69 CRT candidates undergoing DSE, Ciampi et al. showed that the presence of contractile reserve defined by a wall motion score index variation >0.20 is associated with significant LV reverse remodelling and survival after CRT delivery (30). Low-dose DSE is also able to disclose/accentuate the presence of visual LV dyssynchrony, making the detection of dyssynchrony much easier at peak stress, and helping the identification of CRT responders (29).

The measure of global LV function parameters at baseline might provide useful information for the prediction of CRT response. Several single-centre studies (31–33, 54) and two recent large metanalyses (55, 56) showed that LV global longitudinal strain measured before CRT delivery is both a predictor of CRT-induced reverse remodelling and prognosis. In 205 HF patients referred for CRT implantation, Delgado-Monteiro et al. showed that a GLS>|−9%| was an independent predictor of a composite endpoint including death, circulatory support implantation and heart transplantation (HR: 2.91; 95% CI: 1.88–4.49; *p* < 0.001). Interestingly, the predictive value of this cut-off was maintained in patients with intermediate QRS width (120–150 msec), thus helping to refine the selection of patients with a class II indication for CRT implantation (31). The analysis of 1,185 patients from the Leiden CRT registry showed that some subjects can present a discordant CRT response, defined as an improvement in LVESV (left ventricular end-systolic volume) or CRT. The absence of increase in GLS despite the positive LV reverse remodelling might be attributed to an effective resynchronization of the LV ventricle, without concomitant increase in the contractile reserve. Patients with concomitant improvement in LVESV and CRT had the best prognosis [hazard ratio (HR) 0.47; 95% CI: 0.31–0.71, *p* < 0.001], whereas patients with a discordant improvement showed an intermediate survival benefit from CRT (33) (Table 1).

### 3.5. Assessment of scar extension and localisation

In patients with ischemic cardiomyopathy, the extension and localisation of myocardial scar significantly impact CRT response essentially in two ways: (1) the LV lead placement at areas of scar is associated with poor clinical and echocardiographic response to CRT (34, 35); (2) the higher is the scar burden, the lower is the residual LV contractility (10, 30).

As a matter of fact, in the Multicenter Automatic Defibrillator Implantation Trial with Cardiac Resynchronization Therapy (MADIT-CRT), ischemic cardiomyopathy emerged as an independent predictor of LV remodelling after CRT (57). Moreover, ischemic aetiology is associated with a worse prognosis and higher rate of HF hospitalisation in CRT candidates (58).

Cardiac imaging can have a pivotal role in the assessment of myocardial scar. In the landmark randomized TARGET trial, Khan et al.; showed that the positioning of the LV lead in a site far from LV scar and with significant residual contractility (defined as a >10% amplitude of the corresponding radial strain trace) was associated with a higher percentage of CRT response (70 vs. 55%, *p* < 0.031) and a significantly better survival (log rank *p* = 0.002). Nevertheless, when compared with scar assessment at cardiac magnetic resonance (CMR), the predictive value of segmental radial strain for the identification of myocardial scar is low (sensitivity 33% and specificity 72%) (59), suggesting the usefulness of a multimodality imaging approach to plan and ease CRT implantation.

Specifically, echocardiography can assess mechanical dyssynchrony, whereas CMR can quantify the extent and localization of myocardial scar.

Aalen et al. have shown that the localization of myocardial scar in the septum or in the lateral wall can impact LV dyssynchrony patterns at strain echocardiography (10).

If CMR is not available or contraindicated, SPECT might be applied for the localization of the scar and to assess viability (36, 51). In CRT candidates, the quantification of scar burden by SPECT has been associated with CRT response and prognosis (37). However, in the presence of normal coronary artery and LBBB, SPECT can display perfusion defects in the septal and apical segments. These perfusion abnormalities are due to the relative septal hypoperfusion, compared to the lateral wall and can be disclosed by the measure of the absolute regional myocardial perfusion at positron emission tomography (60).

Computed tomography (CT) can be used to analyse the individual's coronary venous anatomy and identify the best pacing site. Moreover, CT might be an alternative to CMR or SPECT to evaluate myocardial perfusion and scar, especially when these other imaging modalities are not available (61).

Despite the interesting perspectives provided by multi-modality (MMI) in CRT (62), 2 large randomized trials applying a combination of echocardiography, CMR and CT did not show any survival benefit of the multi-modality imaging-derived approach vs. the standard approach for CRT implantation (63, 64). Nevertheless, these studies showed that patients receiving the LV lead in the optimal site had better LV remodelling and survival after CRT, supporting the usefulness of a multimodality imaging approach at least in selected cases.

In addition, both these studies focused on the localisation of the myocardial scar in the lateral wall and neglected the value of septal scar and of the septal-to-lateral wall interplay that has shown to be a key element to understand the pathophysiology of LBBB and predict CRT response and prognosis (8, 10).

**Figure 4:**
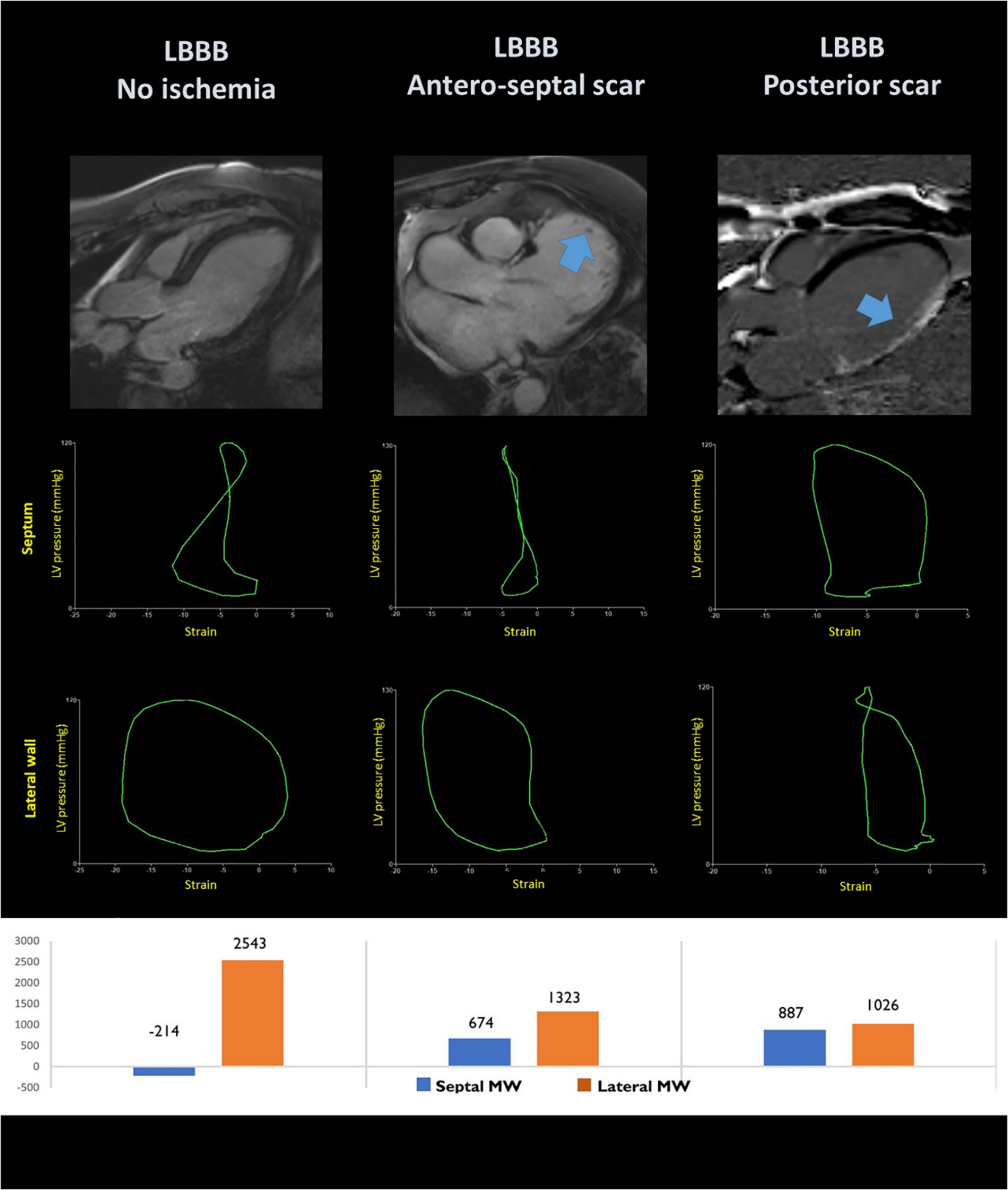
Pressure-loops analysis in the septal and lateral segments of patients with left bundle branch block without ischemic heart disease (left panel); with anteroseptal scar (central panel) and lateral wall scar (right panel). The corresponding localisation of the myocardial scar is shown at MRI late gadolinium enhancement in the upper panels.

In 200 CRT recipients, the septal-to-lateral wall difference in myocardial work, emerged as an independent predictor of CRT response with area under the curve (AUC) of 0.77 (95% CI: 0.70–0.84). Nevertheless, the combination of work difference and septal viability increased the AUC to 0.88 (95% CI: 0.81–0.95). Interestingly, the predictive power of a combination of work assessment and septal scar was superior to QRS width for the prediction of LV remodelling after CRT and was an independent predictor of prognosis HR (0.21, 95% CI: 0.072–0.61) (8). These results underscore the importance of a combination of viability and septal-to-lateral wall dynamics for the evaluation of CRT candidates (Figure 4 and Table 1).

## 4. The importance of a comprehensive evaluation of cardiac function

The imaging-driven analysis of CRT candidates is substantially focused on the assessment of LV function. Nevertheless, an increasing amount of literature shows the importance of a comprehensive approach when imaging CRT candidates for both the identification of CRT-responders and prognostic stratification.

The presence of an extensive LV remodelling before CRT delivery, with more severe LV dysfunction and significant LV dilatation is associated with poor prognosis and to less LV ejection fraction improvement (38, 39).

The assessment of diastolic function is particularly challenging in CRT candidates. Despite the application of the algorithm proposed by Nagueh et al. is discouraged in patients with LV conductions disturbances (65), Andersen OA et al. have shown that this approach is able to identify patients with elevated LV filling pressure against the invasive gold standard also in the case of LBBB or paced rhythm (66).

Previous studies have shown that CRT doesn't influence LV relaxation and successful CRT does not seems to impact the prevalence of diastolic dysfunction (DD) in CRT responders (40, 67). Nevertheless, the degree of DD in CRT candidates is an independent predictor of mortality (HR 6.04; 95% CI: 2.32–15.77 and HR 4.64, 95%CI: 1.49–14.39 for grade II and III DD, respectively) and unsuccessful CRT is associated with an increased prevalence of grade III DD (40).

The left atrium (LA) reflects the cumulative effect of LV filling pressure over time. In the MADIT-CRT trial, CRT was associated with a significant reduction of LA volume, which portended a substantial reduction in atrial tachyarrhythmias (68). In the same cohort, LA indexed volume >52 ml/m^2^ and the lack of LA remodelling after CRT were both associated with a higher hazard of HF and mortality (41). Together with the assessment of LA size, the evaluation of LA reservoir strain (LARS) by speckle tracking echocardiography provides information on the effects of CRT on the LA. CRT is associated with a significant improvement in LARS in responders (42, 69) and baseline LARS is and independent predictor of both LV systolic and diastolic remodelling at 6-month follow-up (*r* = −0.14, *p* = 0.049, *r* = −0.17, *p* = 0.002, respectively (42).

Because the main determinants of LARS are diastolic function and LV longitudinal function, LARS might allow a comprehensive assessment of both the systolic and diastolic LV impairment: the more impaired LARS, the more advanced is the ongoing left ventricular disease, and the less likely is CRT-induced reverse remodelling.

Finally, the relationship between RV function and left ventricular remodelling after CRT is object of debate. In a large meta-analysis of 16 studies, including 1,764 patients, Sharma et al. underscored that baseline RV function as assessed by tricuspid annular plane systolic excursion (TAPSE), RV fractional area change (FAC), RV strain or RV ejection fraction does not determine response to CRT as assessed by change in LVEF (70). Nevertheless, more recent papers question these results by showing that RV dysfunction is predictor of poor prognosis in CRT candidates (43, 44). Interestingly, in an experimental model of LBBB Storsen et al. observed that LBBB causes an abnormal RV free wall motion pattern that is reversed after successful CRT only in the case of preserved RV function (71).

**Figure 5:**
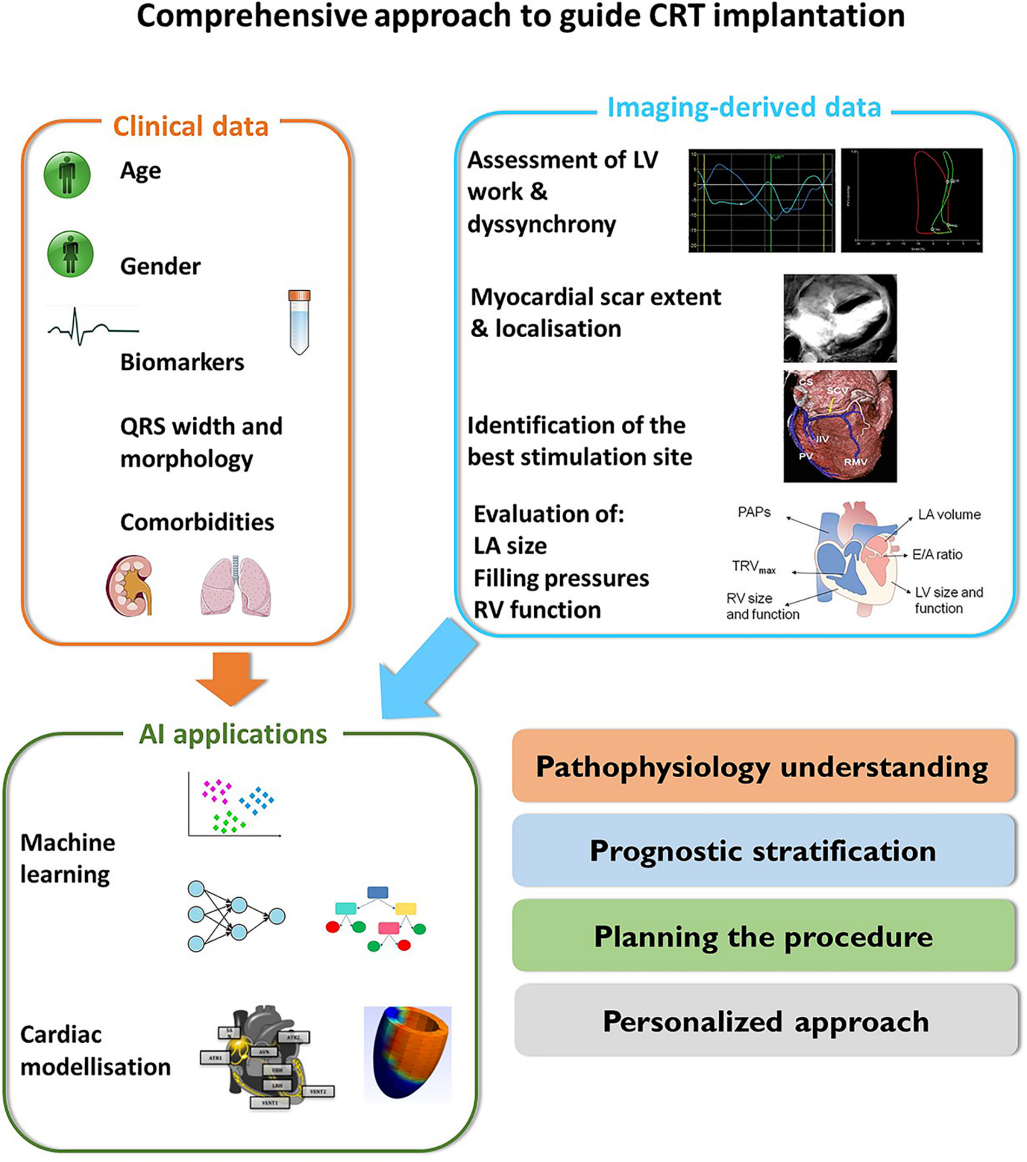
A comprehensive approach to guide CRT implantation.

This might be attributable to the LV/RV interdependence through the LV septum, but can also be influenced by the effect of severe LV dysfunction on mitral regurgitation, filling pressure, tricuspid regurgitation and pulmonary pressure (72) (Figure 5 and Table 1).

## 5. Potential applications for artificial intelligence in CRT

Artificial intelligence (AI) is focused on the application of computational algorithms to huge amounts of data to identify patterns between variables that are not disclosed by the application of standard statistical methods (73). This approach seems particularly interesting in the field of CRT, where the high heterogeneity of CRT-candidates is probably the main determinant of the well-known wide variability in clinical benefit and outcomes (74).

In 154 CRT candidates, our group showed that the application of a random forest method (RF) and Monte Carlo cross validation allowed the identification of 6 strain-derived variables that were able to predict CRT-response with an AUC of 0.80 (95% CI: 0.77 to 0.94) (75).

By the analysis of a larger multicentric population of 323 CRT-candidates, Gallard et al. strengthened previous results and demonstrated that the application of a multifeatured learning method including visual LV dyssynchrony, standard echocardiographic parameters and strain-derived data was able to improve the prediction of CRT-response compared to the simple analysis of QRS duration, with an overall AUC or 0.81 (76).

Moreover, the semi-automatic analysis of strain traces and the subsequent application ML algorithms might be helpful to disclose relevant information that are not evident from the simple visual analysis on strain curves. The application of different methods such as out-of-bag random forest, wrapping and filtering to strain traces obtained in 4-, 3- and 2-chamber views has shown that the most important features are calculated from the 4-chamber view, essentially from the analysis of the antero-septal and basal-septal segment (77).

The application of an unsupervised ML algorithm to 1,106 HF patients from the MADIT-CRT trial was able to identify 4 phenogroups of patients, having different CRT-response and prognosis (78). Interestingly, the analysis of left atrial, LV and right ventricular morphology and function added significant information for the classification of patients. Using both supervised and unsupervised ML methods, Galli et al. were able to underscore the value of right ventricle-derived parameters for the prediction of CRT response and survival. This approach was able to reliable predict CRT response and outcome with an AUC or 0.81 (95% CI: 0.74–0.87) and 0.84 (95% CI: 0.75–0.93), respectively (79).

Similarly, the application of a clustering approach (k-means method and Gaussian mixture model) to clinical and CMR-derived features was also able to stratify CRT-candidates in three survival groups. Interestingly, the addition of CRT-volumetric response to selected pre-CRT data significantly increased the performance of the model for the prediction of prognosis (AUC 0.78 ± 0.04 vs. 0.86 ± 0.02) (80).

Although it is difficult to directly compare classifier performances because different datasets and ML models were used in these studies, these results show the added value of multivariate analysis for the prediction of CRT response.

Computer models represents another interesting application to merge imaging and ECG-derived data to drive computer simulation for the pathophysiological understanding of left ventricular dyssynchrony, improve response to CRT, and for the planning of CRT delivery. These models range from the simple bidimensional representation of LV function (81, 82), to complex 3D-models including the analysis of the molecular, electrical and mechanical properties of the myocardium (83, 84).

Moreover, the combination of clinical and model-derived data might contribute to enhance the pathophysiology understanding of CRT and also increase the accuracy in the prediction of LV remodelling and prognosis in CRT candidates (85).

The computational modelling of LV dyssynchrony relies on the merge of several different competences from imaging and electrophysiology to numerical analysis (Figure 5). The main challenge in this field is the large need of clinical validation, followed by the production of user-friendly tools that allow the analysis of data in a clinically useful time frame (86). The final goal is to merge clinical data and computer simulations to develop digital twins that are able to replicate the specific patient's heart disease and simulates the effect of CRT delivery (87) (Table 2).

**Table 2:** Summary of the main cited studies providing an overview of the role of machine learning in the field of CRT.

Authors/Journal – year/Ref	Population	Endpoints	Methods	Parameters	Results
Donal E et al. *JACC Cardiovasc Imaging 2019.* (75)	154 pts LVEF < 35%, QRS width > 120 msec	LVESV reduction >15% at 6 months FU	Random Forest and Monte Carlo cross-validation	60 features from semi-automatic strain trace analysis, QRS width, visual Lv dyssynchrony	Extraction of 6 main features with an AUC 0.804 (0.77–0.94) for the prediction of LV reverse remodelling
Gallard A. et al. *Int J Cardiovasc Imaging 2021.* (76)	221 pts LVEF < 35%, QRS ≥120 msec	LVESV reduction >15% at 6 months FU	Random Forest	Features from echocardiography, strain traces analysis, QRS width, LV dyssynchrony	The combination of SF, E, E/A, E/e′, QRS width, LVESV and 8 features obtained from strain curves predicted LV reverse remodelling with an AUC of 0.81 ± 0.05
Gallard A et al. *PloS One 2021.* (77)	161 pts LVEF < 35%, QRS width > 120 msec	LVESV reduction >15% at 6 months FU	Random Forest	158 features from semi-automatic strain trace analysis, QRS width, LVEF	20 main features from strain analysis are the best predictors of LV reverse remodelling. 50% of these features are derived from the 4-chamber strain.
Cikes M et al. *Eur J Heart Fail 2019.* (78)	1,106 LVEF ≤ 30%, QRS width ≥130 ms	Overall death or HF hospitalisation	Multiple Kernel Learning and K-means clustering	Four phenogroups	Two phenogroups with better prognosis (HR = 0.35, 95% CI: 0.19–0.64, *p* < 0.0001 and HR = 0.36, 95% CI: 0.19–0.68, *p* = 0.001)
Galli E et al. *J Am Soc Echocardiogr 2021*. (79)	193 pts LVEF 29 ± 8%, QRS width 167 ± 21, 33% ischemic	Primary: heart transplantation, LV-assisted device implantation overall death	k-medoid, Boruta algorithm, random forest	28 clinical, biological and echo-derived variables; two phenogroups	Prognostic value of the main 8 variables: AUC 0.84 (0.75–0.93); Two phenogroups with different prognosis: HR = 4.70, 95% CI: 2.1–10.0, *p* < 0.0001; log-rank *p* < 0.0001)
Bivona DJ et al. *Heart Rhythm 2022.* (80)	200 pts LVEF 24.0 (17.7–30.5)%, QRS 58 (142–175) msec, 43.5% ischemic	1) Reduction of LVESV at 6 months FU 2) Survival	k-means method and Gaussian mixture model	39 features derived from CMR and biology	3 clusters able to predict CRT response and prognosis

AUC, area under the curve; CI, confidence interval; CMR, cardiac magnetic resonance; FU, follow-up; HF, heart failure; HR, hazard ratio; LV, left ventricle; LVEF, left ventriculare ejection fraction; LVESV, left ventricular end-systolic volume.

The application of AI in medicine sounds appealing in several clinical scenarios including CRT because of its intrinsic capacity of analysing a huge amount of heterogeneous data and providing useful outcomes for diagnosis, management and prognosis. AI might therefore provide a fundamental contribution to the development of precision medicine and to a personalized approach to patients' care (88). However, several concerns exist about the wide application of AI in the health care system. These issues goes from the quality and transparency of data, to complex technical issues, to ethical and political concerns and need to be solved to provide a safe and fair application of AI in the field of medicine (73, 89).

## Conclusions

Cardiac resynchronization therapy has a pivotal role for the management of patients with systolic heart failure and wide QRS. In the recent years, the excessive relevance given to the concept of “non-response” to CRT seems to contribute to the substantial underuse of the device, so that up-to two third of patients needing the CRT according to recommendations are not implanted (90).

This review provides an insight on the progresses of imaging in the field of CRT and underscore the need of a comprehensive approach which is useful to (1) disclose the electromechanical substrates more suitable to respond to CRT; (2) identify imaging pattern that are associated with good CRT response also in patients with unclassical indications; (3) emphasize the relevance of a global assessment of cardiac function including the evaluation of filling pressure, LA size and function, and RV function; (4) underscore the potentiality of AI to merge clinical, electrophysiological and imaging-derived data with the purpose of proposing patient's tailored strategies.

The final goal of this holistic approach to CRT is to individualize the treatment and to optimize CRT delivery.
